# Chronic myeloid leukemia‐derived extracellular vesicles increase Foxp3 level and suppressive activity of thymic regulatory T cells

**DOI:** 10.1002/eji.201848051

**Published:** 2019-12-05

**Authors:** Julian Swatler, Wioleta Dudka, Lukasz Bugajski, Marta Brewinska‐Olchowik, Ewa Kozlowska, Katarzyna Piwocka

**Affiliations:** ^1^ Laboratory of Cytometry Nencki Institute of Experimental Biology Polish Academy of Sciences Warsaw Poland; ^2^ Department of Immunology Faculty of Biology University of Warsaw Poland

**Keywords:** chronic myeloid leukemia, extracellular vesicles, Foxp3, immunosuppression, regulatory T cells

## Abstract

Mechanisms driving immunosuppression in chronic myeloid leukemia are mostly unknown. We show that leukemic extracellular vesicles (EVs) target lymphocytes and amplify suppressive function of thymic regulatory T cells, by driving expression of Foxp3 transcription factor. This could facilitate expansion of leukemic cells outside the bone marrow, leading to blast crisis.

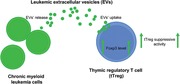

##       

Extracellular vesicles (EVs), including exosomes and microvesicles, are one of the key factors controlling immune function, both in steady state and pathological conditions 1. Various studies have exemplified their role in driving regulatory T cells (Tregs) differentiation and suppressive activity in solid tumors, contributing to tumor‐promoting immunosuppression 2, 3. BCR‐ABL‐positive chronic myeloid leukemia (CML) immune signature has only recently been studied in detail 4, 5 and CML has also been recognized as an immunosuppressive malignancy. Elevated amount of Foxp3+ regulatory T cells in blood and bone marrow (BM) of CML patients 4, 5 suggests their role in disease development. However, very little is known about mechanisms that drive the immunosuppressive signature of CML 6. We hypothesize that chronic myeloid leukemia‐derived EVs can be a contributing factor, due to their transfer and presence also in distant tissues outside the CML bone marrow niche.

Our data presented here strongly indicate that CML‐derived EVs can regulate Foxp3 expression and suppressive activity of Foxp3+ thymic regulatory T cells (tTregs). To study effect of CML‐derived EVs specifically on the tTregs population, we have sorted tTregs directly from murine thymi (Supporting Information Fig. 1), and confirmed their thymic identity/origin by assessment of Helios marker (Supporting Information Fig. 2).

tTreg cells were treated with leukemic EVs isolated from conditioned media of murine 32D progenitor cells expressing BCR‐ABL (32D BCR‐ABL+), as CML model, or the parental 32D cells. EVs were characterized based on their morphology, size, and expression of specific markers, according to the guidelines of ISEV (International Society for Extracellular Vesicles) and the EV‐TRACK consortium 7, 8. Enrichment of proteins associated with multivesicular bodies (Tsg101, Alix; Fig. 1C) and size of around 100 nm (Fig. 1A and B) indicate that the obtained extracellular vesicles are mainly enriched in exosome fraction. Contamination with mitochondrial or ER components was excluded by the absence of TOM20 and Grp78 proteins (Fig. 1C). We observed strong, dose‐dependent association/uptake of CFSE‐labeled leukemic EVs by different subsets of thymocytes (Fig. 1D and E) – providing an indication that CML‐derived EVs might potentially modulate thymocytes’ function.

**Figure 1:**
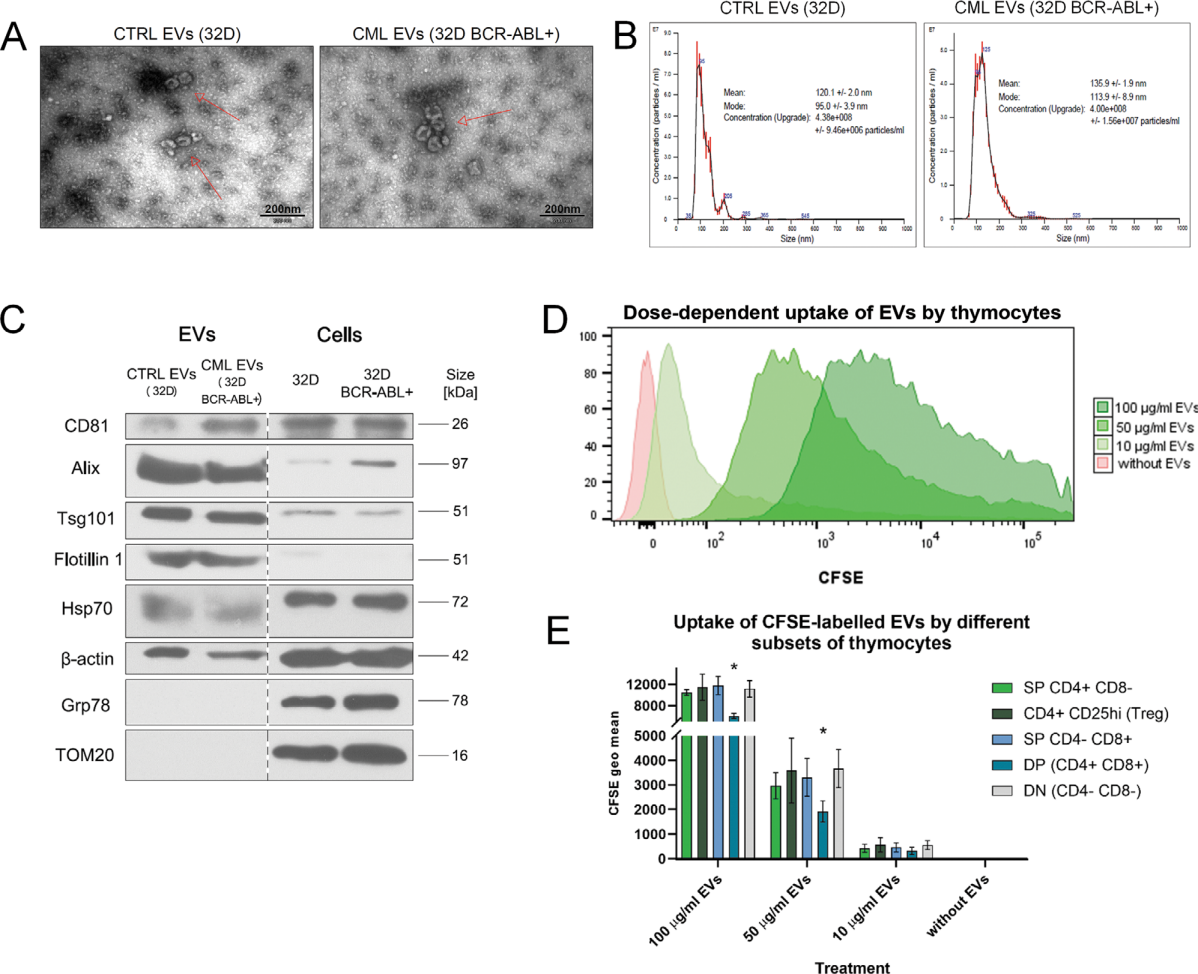
Quality control and characterisation of isolated extracellular vesicles. (A) Representative electron microscopy images of EVs (red arrows) isolated from conditioned media of 32D and 32D BCR‐ABL+ cells. TEM imaging was performed for two independent experiments of EVs’ isolation (each cell line). Scale bar, 200 nm. Widefield images are presented in Supporting Information Fig. 3. (B) Representative nanoparticle tracking analysis of EVs. For each cell line, five to six independent experiments of EVs’ isolations were performed. (C) Western blot analysis of proteins in EVs: enriched (CD81, Alix, Tsg101, Flotilin1), present (Hsp70, actin), and absent (ER marker, Grp78; mitochondrial marker, TOM20). Equal amount of protein was loaded on gels. Data from the same gels are presented (marked with dashed line). For each cell line, three independent experiments of EVs’ isolations were performed, and each protein was assessed on one to two independent western blots (two to four protein markers on each). (D) Representative histograms (two independent experiments, one to two replicates each) of uptake of CFSE‐labeled CML EVs by live thymocytes in vitro. (E) Quantified EVs uptake by different subsets of thymocytes. Data presented as mean ± SD from two independent experiments, two to three replicates each. ^*^ *p* < 0.05, statistical significance between DP and all other subsets (unpaired *t*‐test with Welch's correction). Full gating strategy for (D) and (E) is presented in Supporting Information Figure 4.

Influence of CML‐derived EVs on suppressive properties of tTregs was studies in a modified in vitro suppression assay 9, where tTregs were pre‐incubated with CML‐derived EVs. tTregs treated with CML‐derived EVs exhibited higher suppressive activity toward CD8^+^ responder cells, as demonstrated by two parameters – increased percentage of inhibition by tTregs and decreased expansion index of CD8^+^ responder cells (Fig. 2A and B). Surprisingly, in this setting, no difference was observed in suppression toward CD4^+^ responder cells (Supporting Information Fig. 5). To exclude that differences derive from influence of EVs on responder cells (rather than tTregs), we performed control experiments without tTregs. These confirmed that the difference in suppressive effect is due to strong influence of CML EVs on tTregs’ activity, as weaker proliferation of CD8^+^ cells was not observed when tTregs were lacking in the cell culture, and CML‐derived EVs even amplified proliferation of responder cells alone (Supporting Information Fig. 6). To confirm that increased proliferation of responder cells does not constitute a dominant effect in a more physiological setting, tTregs, responder lymphocytes and EVs were cultured together from the beginning in an in vitro suppression assay. Also in this setting, tTregs exhibited increased inhibitory function in the presence of CML‐derived EVs (Fig. 2C; Supporting Information Fig. 7), however, toward both CD8^+^ and CD4^+^ lymphocytes. Nevertheless, slightly stronger suppression toward CD8^+^ than CD4^+^ responder cells was observed, further suggesting possible preferential suppression toward CD8^+^.

**Figure 2:**
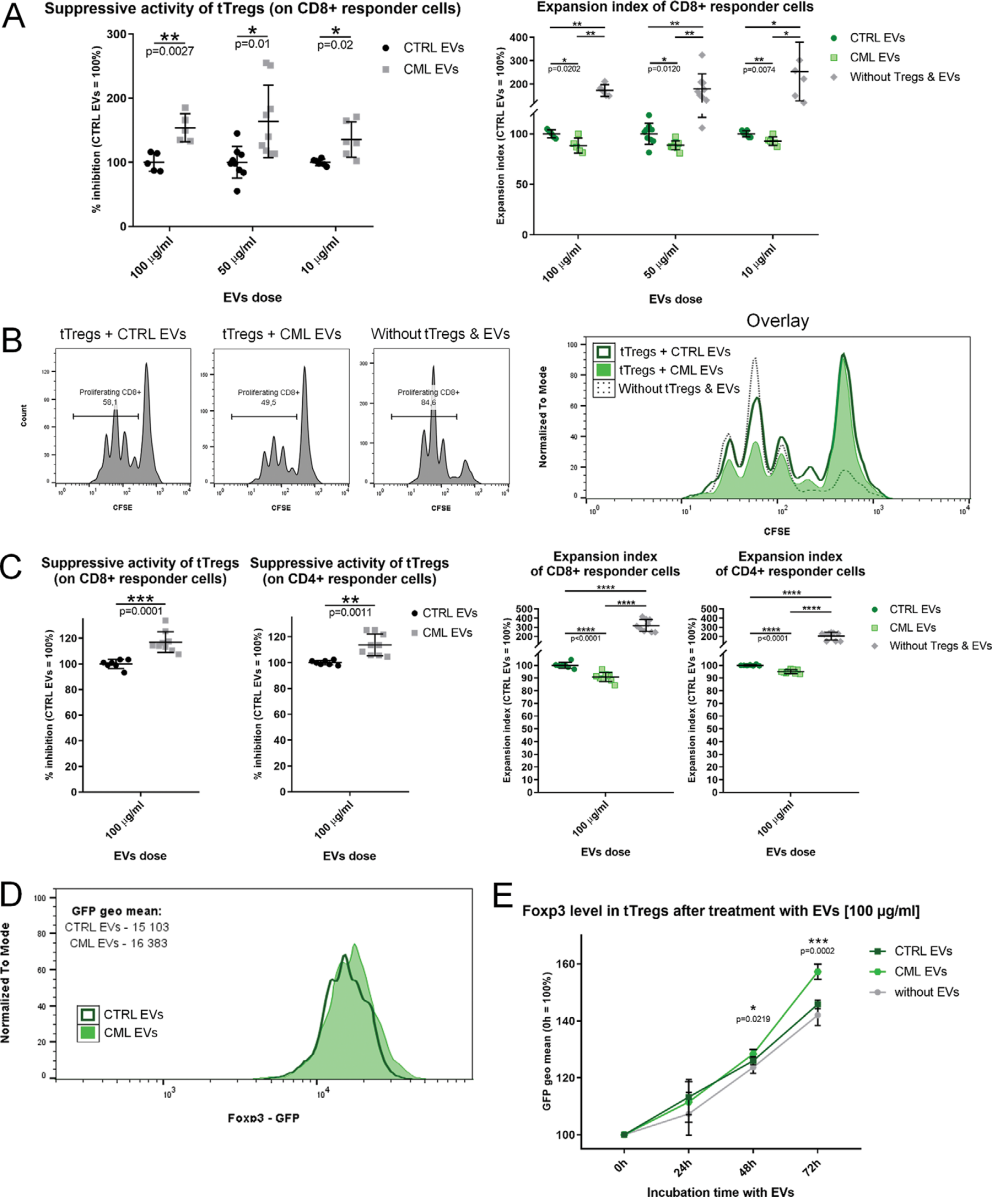
CML‐derived EVs increase Foxp3 expression and suppressive function of tTregs. (A) Suppressive activity (left) of tTregs toward CD8^+^ responder cells after treatment with CTRL/CML EVs. Expansion index (right) of CD8^+^ responder cells in culture with tTregs treated with CTRL/CML EVs. Increase in suppressive activity corresponds to decrease in expansion index. Data normalized to the average values in samples treated with CTRL EVs are presented. Single data points with mean ± SD are presented. ^*^ *p* < 0.05; ^**^ *p* < 0.01 (unpaired *t*‐test with Welch's correction). (B) Representative histograms (individual and overlay) of CD8^+^ responder cells proliferation in cultures with EVs‐treated [50 μg/mL] tTregs. (C) Suppressive activity (left) of tTregs and expansion index (right) of responder cells in a combined culture of tTregs, responder lymphocytes, and EVs. Data normalized to the average values in samples treated with CTRL EVs are presented. Single data points with mean ± SD are presented. ^**^ *p* < 0.01, ^***^ *p* < 0.001, ^****^ *p* < 0.0001 (unpaired *t*‐test with Welch's correction). For (A–C), data from three independent experiments (two to three replicates each) were presented. (D) Representative Foxp3 expression (in cells co‐expressing Foxp3 and EGFP) in tTregs treated with CTRL/CML EVs. (E) Quantified Foxp3 expression in tTregs treated with CTRL/CML EVs or untreated with EVs. Data were normalized to Foxp3 expression at 0 h. Mean ± SD is presented. ^*^ *p* < 0.05, ^***^ *p* < 0.001 (unpaired *t*‐test with Welch's correction) – significance between CML and CTRL EVs. For (D) and (E), data from three independent experiments (one to two technical replicates each) were presented. Full gating strategy for (A–C), (D), and (E) is presented in Supporting Information Figures 8 and 9.

Regulation of cellular function by different types of extracellular vesicles is still largely unknown. Thus, identification of cellular components and processes targeted by EVs is of high interest. Regulatory T cells can exert suppressive activity through multiple molecules – surface receptors (e.g., CTLA‐4, LAG‐3), cytokines (TGF‐β, IL‐10, IL‐35), or ectoenzymes CD39 and CD73, which in majority are either directly or indirectly regulated by Foxp3 transcription factor 10. We found that treatment of tTregs with CML‐derived EVs leads to increase of Foxp3 level (Fig. 2D‐E). This demonstrates that leukemic EVs target and control tTregs largely by increasing expression of central factor regulating Tregs’ biology – Foxp3.

In summary, we demonstrate that CML‐derived extracellular vesicles upregulate Foxp3 expression in thymic regulatory T cells, leading to increased suppressive activity of these cells, preferentially (to some extent) toward CD8^+^ responder cells. This may seem crucial for immunosuppression in CML, as cytotoxic CD8^+^ T cells constitute one of the main lines of immune system defense against leukemia 6. Although potentially interesting, this observation needs deeper mechanistic studies. Biology of tTregs in leukemia is poorly understood, but with multiple overexpressed self‐proteins and high prevalence of self‐antigens in CML cells tTregs are of high significance 6. As thymic Tregs (tTregs) are responsible for tolerance to autologous cells and self‐antigens, they can thus suppress anti‐leukemic immunity by conferring tolerance to specific leukemic antigens recognized as self. Control of tTreg cells by leukemic EVs presents a novel mode of promoting immunosuppression in CML, but also in cancer in general. Importantly for chronic myeloid leukemia, EVs could target distant immune cells outside the CML close microenvironmental niche, thus inducing widespread immunosuppression and facilitating expansion of leukemic cells outside the bone marrow, promoting disease progression. Further dissection of described phenomenon is necessary to identify specific components in EVs that regulate Foxp3 expression, as well as identify functional changes in Tregs (both thymic and peripheral), especially in terms of direct and indirect interactions with other subsets of immune cells. Altogether, this may lead to development of novel prognostic markers and targets for immunotherapy in leukemia.

## Conflict of interest

The authors declare no commercial or financial conflict of interest.

**Abbreviations**
CML: chronic myeloid leukemia
EVs: extracellular vesicles
Foxp3: forkhead box P3
Tregs: regulatory T cells
tTregs: thymic regulatory T cells

## Supporting information

Supporting information
