# Supplementary material for: Chronic myeloid leukemia‐derived extracellular vesicles increase Foxp3 level and suppressive activity of thymic regulatory T cells
Source: Eur J Immunol. 2019 Dec 5;50(4):606–9. doi: 10.1002/eji.201848051 (PMC7187374; doi:10.1002/eji.201848051)
Supplement: Supplementary file 1 — Supporting information [file EJI-50-606-s001.pdf]

MATERIALS AND METHODS

Cell culture

32D mouse progenitor cells and 32D BCR-ABL-expressing cells (32D BCR-ABL+) were a kind gift from Dr. S. L. McKenna and were cultured as described (Wolczyk *et al*., BBA, 2016). Lymphocytes were cultured in RPMI 1640 medium with 10% heat-inactivated fetal bovine serum (Gibco), 50mU/ml penicillin (Gibco), 50ng/ml streptomycin (Gibco), 0.2mM 2-mercaptoethanol (Gibco) and 50U/ml IL-2 (Peprotech).

Extracellular vesicles isolation

Extracellular vesicles (EVs) were isolated by differential centrifugation (Théry *et al*., Curr Protoc Cell Biol, 2006) from conditioned medium of 32D and 32D BCR-ABL+ cells. Briefly, cells were seeded at density of 0.4 x 10^6 cells/ml for 24 hours prior to media collection. Cell suspension and supernatants were first centrifuged at 160 x g, 320 x g and 1300 x g, to remove live and apoptotic cells. Afterwards, medium was subjected to differential ultracentrifugation – 40 minutes at 15,000 x g (4°C) (to remove debris and larger vesicles) and afterwards for 100 minutes at 140,000 x g (4°C), to pellet EVs. Pelleted EVs were then washed in PBS (100 minutes, 140,000 x g, 4°C). Final pellet was resuspended in non-supplemented RPMI medium for functional assays or in PBS for EVs characterisation. Ultracentrifugation was performed using 45Ti rotor and Optima XPN-100 ultracentrifuge (Beckman Coulter).

Transmission electron microscopy

Isolated EVs, resuspended in PBS, were deposited on formvar-coated copper grids (for 20 minutes), fixed in 2% PFA (20 minutes) and contrasted with 4% uranyl acetate (for 10 minutes, on ice, in the dark). After each step, residual liquid was blotted on filter paper. Obtained preparations were observed using transmission electron microscope JEM 1400 (JEOL Co., Japan, 2008) at Laboratory of

Electron Microscopy, Nencki Institute, under 60kx (widefield view) or 150kx (close-up view) magnification.

Western blotting

To obtain protein lysates, EVs or cells were lysed at 95 °C in a buffer containing: 50 mM Tris-HCl pH 6.8, 10% glycerol and 2% SDS. Protein concentration was measured by Bradford assay. Equal amounts of protein from EVs and cells were loaded on gels. Primary antibodies used for protein detection were: mouse anti-CD81 (Abcam, #ab79559, 1:500), mouse anti-Alix (Abcam, #ab117600, 1:400), mouse anti-Tsg101 (Santa Cruz, #sc-7964, 1:400), mouse anti-Flotillin-1 (BD, #610820, 1:500), mouse anti-Hsp70 (Abcam, #ab2787, 1:400), mouse anti-Grp78 (BD, #610978, 1:1000), rabbit anti-TOM20 (Santa Cruz, #sc-11415, 1:2000), mouse anti-actin (conjugated with horseradish peroxidase, Sigma-Aldrich, #A3854) and secondary antibodies conjugated with horseradish peroxidase: goat anti-mouse (Dako, #P0447) and goat anti-rabbit (Dako, #P0448). The SuperSignal West Pico ECL (Thermo Fisher Scientific) reagent was used for signal detection.

Nanoparticle tracking analysis (NTA)

NTA measurement enables quantification and size assessment of different vesicles based on detection of Brownian motion in liquid suspension of particles. Freshly isolated EVs, resuspended in PBS, were diluted 1:1000 and analysed using the Nanosight NS300 device.

Uptake of extracellular vesicles

To analyse uptake/association of EVs by thymocytes, EVs from 32D BCR-ABL+ cells were fluorescently labelled with 10µM CFSE (Invitrogen), by adding CFSE to conditioned media before 2nd ultracentrifugation step (100 minutes at 140,000 x g). Residual CFSE was washed out during washing of EVs in PBS (3rd ultracentrifugation step). Specified amounts of EVs were added to previously isolated murine thymocytes (from C57BL/6 mice), that were seeded in 48-well plates at density of 1 x 10^6 cells/ml. After 2 hours of incubation, thymocytes were washed and EVs' uptake was analysed

on BD LSR Fortessa cytometer. For detection of EVs' uptake by specific subsets of thymocytes, cells after *in vitro* culture were stained for viability (eBioscience Fixable Viability Dye eFluor506, Invitrogen) and with specific antibodies to CD4 (APC, BD), CD8 (APC-eFluor780, Invitrogen), CD25 (BV605, BD) and analysed on BD LSR Fortessa cytometer, as presented in Supporting Information Fig. 4. Obtained data was analysed in FlowJo software.

Mice

For presented studies, 8-12 weeks old male mice were used – either of C57BL/6 strain (for suppression assay, due to higher endogenous activity of thymic Tregs, compared to B6.Cg-Foxp3^{tm2Tch} mice) or B6.Cg-Foxp3^{tm2Tch} strain (with C57BL/6 background, that co-express Foxp3 and EGFP, for experiments on Foxp3 level in tTregs). Mice were bred in the Animal Facility at Faculty of Biology, University of Warsaw, under 12/12 light/dark photoperiod, in individually ventilated cage system, with *ad libitum* access to food and water.

All experimental procedures involving mice were performed according to the guidelines of the Poland's National Ethics Committee for Animal Experimentation.

Isolation of cells and sorting

Mice were sacrificed, thymi and axillary lymph nodes were immediately harvested and homogenized in cold PBS. Tissue debris was removed by filtering through 100μm mesh. For sorting of Tregs, thymocytes were stained with fluorochrome-conjugated antibodies to CD4 (APC, BD), CD8 (PE, BD) and CD25 (PE-Cy7, BD) and sorted as presented in Supporting Information Fig. 1 on BD FACS Aria II sorter. Cell sorting was performed according to "Guidelines for the use of flow cytometry and cell sorting in immunological studies" (Cossarizza *et al.*, Eur J Immunol, 2017).

Staining of Helios in sorted Tregs

Tregs from thymi of C57BL/6 (WT) or B6.Cg-Foxp3^{tm2Tch} (Foxp3-EGFP) mice were sorted as presented in Supporting Information Fig. 1. Sorted cells were further washed and stained in deep-well V-bottom

plates. Intracellular staining of Helios in Tregs from C57BL/6 mice was performed using eBioscience Foxp3/Transcription Factor Staining Buffer Set (Invitrogen). In brief, cells were first stained for viability (eBioscience Fixable Viability Dye eFluor506, Invitrogen) and with antibodies to CD4 (APC, BD), CD8 (PE, BD), CD25 (PE-Cy7, BD). After washing, cells were fixed and permeabilized and intracellular proteins were stained with antibodies to Foxp3 (eFluor450, Invitrogen) and Helios (PerCP-eFluor710, Invitrogen). Intracellular staining of Helios in Tregs from B6.Cg-Foxp3^{tm2Tch} mice was performed using Transcription Factor Phospho Buffer Set (BD), as staining with this particular kit does not interfere with fluorescence of GFP. In brief, cell were first stained for viability (eBioscience Fixable Viability Dye eFluor506, Invitrogen). After washing, cells have undergone fixation, permeabilization and additional nuclear membrane permeabilization. Afterwards, surface and intracellular proteins were stained with antibodies to CD4 (APC, BD), CD8 (PE, BD), CD25 (PE-Cy7, BD), Foxp3 (eFluor450, Invitrogen) and Helios (PerCP-eFluor710, Invitrogen). To set up gates for discrimination of Helios-positive cells, FMO-1 controls for Helios-PerCP-eFluor710 were prepared. Cells were analysed on BD LSR Fortessa cytometer. Obtained data was analysed in FlowJo software as presented in Supporting Information Fig. 2. All flow cytometric analyses were performed according to "Guidelines for the use of flow cytometry and cell sorting in immunological studies" (Cossarizza *et al*., Eur J Immunol, 2017).

Assessment of Tregs' suppressive activity, Foxp3 level and proliferation of responder cells *in vitro*

To analyse influence of EVs on suppressive activity of tTregs specifically (Fig. 2A-B, Supporting Information Fig. 5) , sorted CD4+ CD8- CD25hi tTregs were first incubated for 24 hours with specified amounts of EVs. Afterwards, CFSE-labelled responder cells (Tresp, unsorted lymphocytes from axillary lymph nodes) were mixed with Treg at 1:2 Treg:Tresp ratio and stimulated with 0.5µg/ml anti-CD3 and 0.05µg/ml anti-CD28 antibodies (BD) and co-cultured for 72 hours. This experimental setting aimed at maximising EVs' influence on tTreg in a suppression assay culture. Proliferation of responder cells, additionally stained with specific antibodies to CD4 (APC, BD) and CD8 (PE, BD), was

measured by flow cytometric analysis of CFSE dilution, on BD FACS Verse cytometer and analysed in BD FACS Diva and FlowJo software, as presented in Supporting Information Fig. 8. Suppressive activity of Treg was measured as percentage of inhibition (% inhibition = 100 − (% of proliferated cells in co−culture with tTreg)/(% of proliferated cell in control culture without tTreg)x100), expansion index was calculated with proliferation modelling tool in FlowJo.

To analyse influence of EVs on tTregs' suppressive function in a more physiological setting/context and to dissect effect of CML-derived EVs in a wider immune context (Fig. 2C, Supporting Information Fig. 7), sorted tTregs were cultured together with responder T cells and EVs, as well as activated (anti-CD3/CD28), from the beginning of the culture. In brief, sorted CD4+ CD8- CD25hi tTregs were mixed with Cell Proliferation Dye eFluor450-labelled responder cells (Tresp, lymphocytes from axillary lymph nodes) at 1:5 Treg:Tresp ratio (due to culturing of cells together from beginning of the culture, the suppressive effect was stronger and thus Treg:Tresp ration had to be adjusted). Specified amounts of EVs and stimulating anti-CD3 (0.5µg/ml) and anti-CD28 (0.05µg/ml) antibodies were added immediately afterwards and cells were co-cultured for 72 hours. Proliferation of responder cells, additionally stained with specific antibodies to CD4 (APC, BD) and CD8 (PE, BD), was measured by flow cytometric analysis of proliferation dye dilution, on BD LSR Fortessa cytometer and analysed in FlowJo software, as presented in Supporting Information Fig. 7 and 8. Suppressive activity and expansion index were measured and calculated as mentioned above.

For analysis of Foxp3 level in Tregs (Fig. 2D-E), Tregs were first preincubated with EVs for 24 hours and afterwards stimulated with anti-CD3 and anti-CD28 antibodies. Foxp3 level was analysed at the beginning of culture and 24, 48 and 72 hours afterwards. Cultured cells were collected, stained for viability (7-AAD, BD) and with specific antibodies to CD4 (APC, BD), CD8 (PE, BD), CD25 (PE-Cy7, BD) and Foxp3 level was analysed by GFP fluorescence on BD LSR Fortessa cytometer, as presented in Supporting Information Fig. 9.

For analysis of proliferation of responder cells with EVs (Supporting Information Fig. 6), responder cells were cultured as in the suppression assay, only without Treg – to exclude the suppressive effect of EVs themselves on responder cells, as well as largely exclude effects potentially mediated by peripheral/induced Tregs or other cells amongst cells from axillary lymph nodes. In brief, responder cells and EVs were kept in culture separately for 24 hours. Afterwards, responder cells were added to EVs and stimulated with anti-CD3 and anti-CD28 antibodies. After 72 hours of culture with EVs, CFSE dilution in cells was analysed on BD LSR Fortessa cytometer, in the same manner as in the suppression assay (Supporting Information Fig. 8).

Statistical analysis

All experiments were performed as at least 3 independent, biological experiments, with 2-3 technical replicates in each. Statistical analysis and graph preparation were performed in GraphPad Prism 7 software. In each graph, mean value +/- SD are presented. Statistical significance was determined by unpaired t test with Welch's correction. The asterisks indicate statistical significance as described: * - $p<0.05$, ** - $p<0.01$, *** - $p<0.001$, **** - $p<0.0001$.

References

[11] Wolczyk, M. et al., Biochim Biophys Acta Gen Subj. 2017. 1861(5 Pt A):1024-1035

[12] Théry, C. et al., Curr Protoc Cell Biol. 2006. Chapter 3:Unit 3.22

[13] Cossarizza, A. et al., Eur J Immunol. 2017. 47(10):1584-1797

SUPPORTING INFORMATION FIGURES

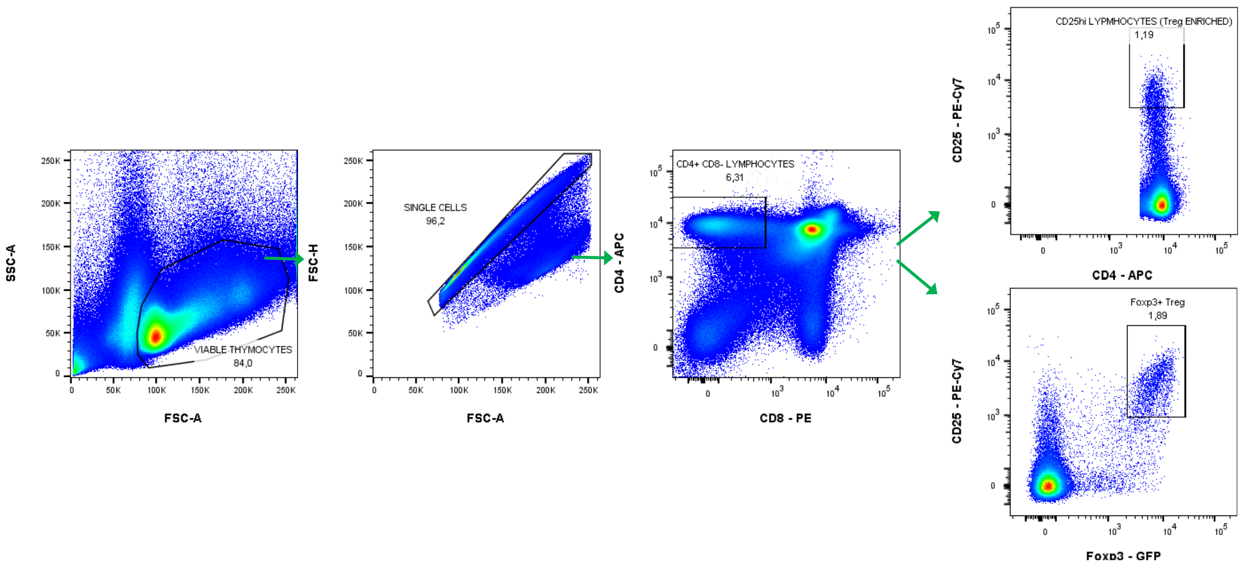

Supporting Information Figure 1. Gating strategy for sorting of tTregs (CD4+ CD8- CD25hi or CD4+ CD8- CD25+ Foxp3+).

Representative demonstration (of 6 independent experiments) of the gating strategy for flow cytometric sorting of tTregs (characterized as either CD4+ CD8- CD25hi or CD4+ CD8- CD25+ Foxp3+) from the thymus. Cells collected from the thymus were stained with fluorochrome-conjugated antibodies against CD4 (APC), CD8 (PE) and CD25 (PE-Cy7), Foxp3 was detected based on GFP signal (in cells from B6.Cg-Foxp3^{tm2Tch} transgenic mice that co express Foxp3 and EGFP). Presented data was analyzed using FlowJo software.

A

Helios expressing tTregs, C57BL/6 mice:

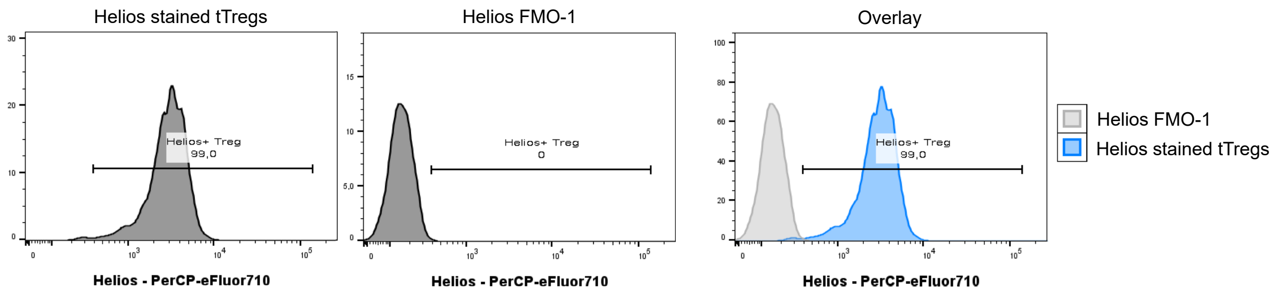

Helios expressing tTregs, B6.Cg-Foxp3[tm2Tch] mice:

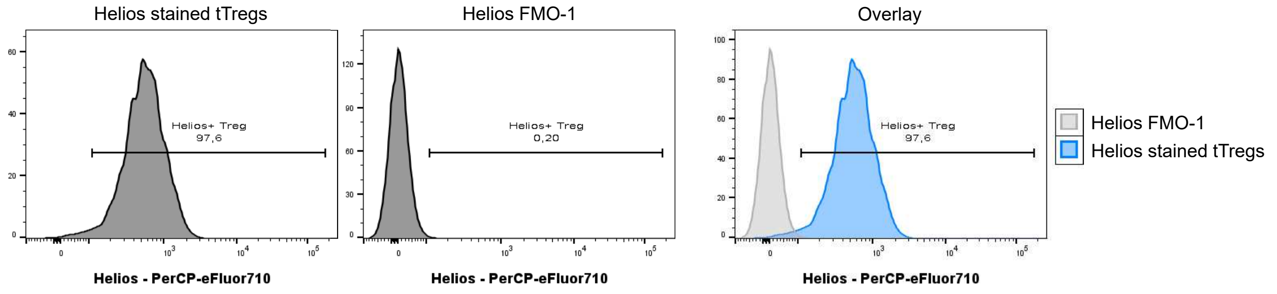

B

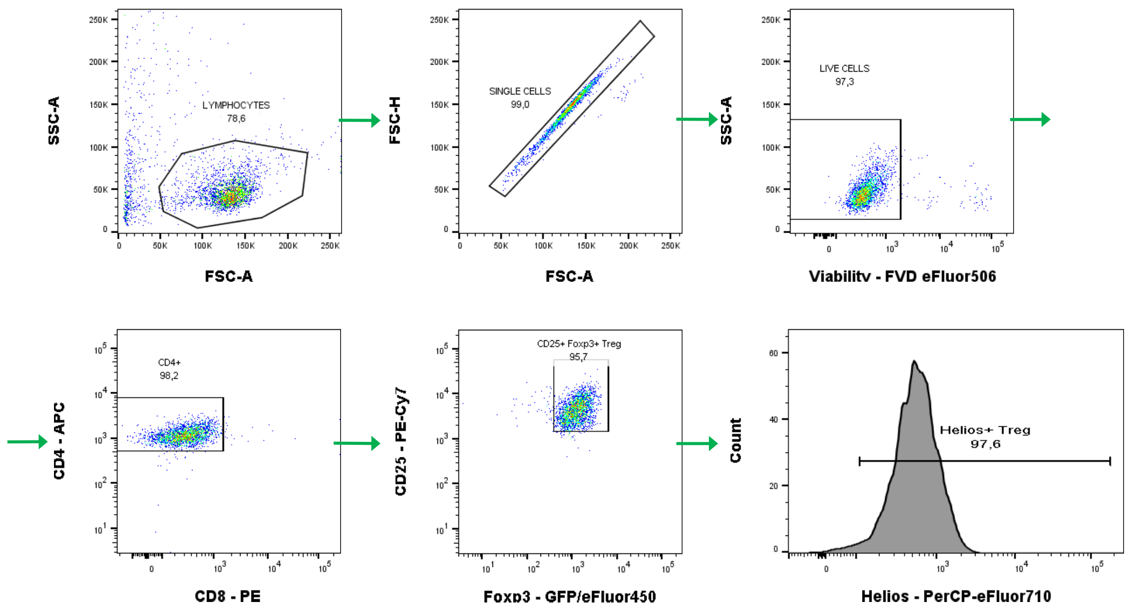

Supporting Information Figure 2. Gating strategy and analysis of Helios expression in sorted tTregs.

A) Representative (of 2 independent experiments for each mouse strain) histograms (individual and overlay) of Helios expression in sorted Tregs from thymus of C57BL/6 or B6.Cg-Foxp3^{tm2Tch} mice. Cells in "CD25+ Foxp3+ Treg" gate are shown on histograms. B) Representative demonstration (of 4 independent experiments) of the gating strategy for flow cytometric analysis of Helios expression in CD4+ CD8- CD25+ Foxp3+ Tregs sorted from the thymus. Sorted cells were stained for viability (with fixable viability dye - FVD eFluor506), surface markers (with fluorochrome conjugated antibodies against CD4 (APC), CD8 (PE) and CD25 (PE-Cy7)) and intracellular proteins (with fluorochrome conjugated antibodies against Foxp3 (eFluor450) and Helios (PerCP – eFluor710)). In cells from B6.Cg-Foxp3^{tm2Tch} mice Foxp3 was detected based on GFP signal. Presented data was analyzed using FlowJo software.

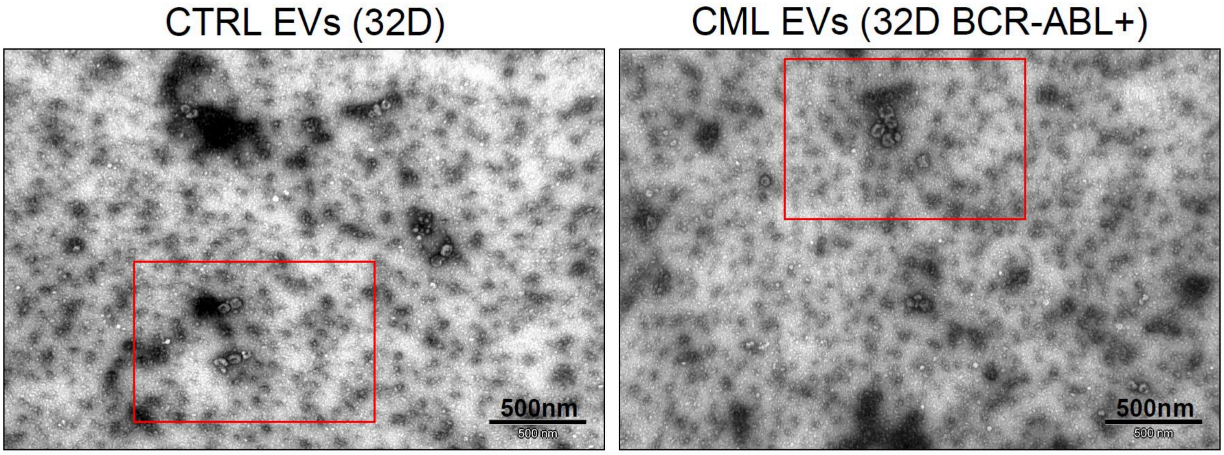

Supporting Information Figure 3. Widefield electron microscopy images of EVs isolated from conditioned media of 32D (CTRL EVs) and 32D BCR-ABL+ cells (CML EVs).

Representative widefield electron microscopy images of EVs isolated from conditioned media of 32D (CTRL EVs) and 32D BCR-ABL+ cells (CML EVs). Scale bar - 500nm. Red frames indicate close-up fields presented in Figure 1A. TEM imaging was performed for EVs from 2 independent experiments of EVs' isolation (for each cell line).

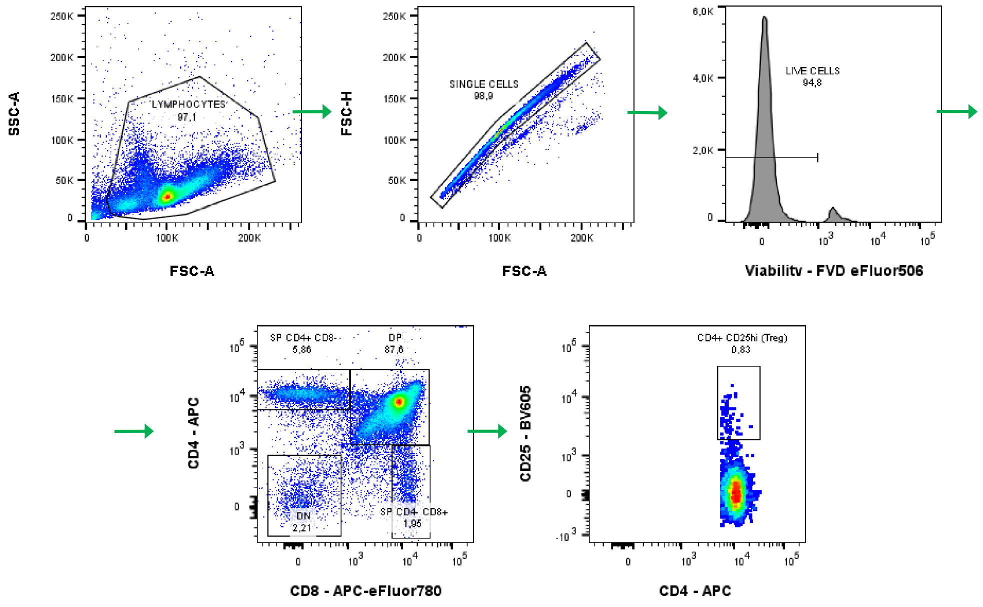

Supporting Information Figure 4. Gating strategy for analysis of uptake of CFSE-labelled EVs by different thymocyte subsets.

Representative demonstration (of 2 independent experiments) of the gating strategy for flow cytometric analysis of uptake of CFSE-labelled EVs by thymocytes. Cells collected from *in vitro* cultures were stained with fluorochrome-conjugated antibodies against CD4 (APC), CD8 (APC eFluor780), CD25 (BV605) and with fixable viability dye eFluor506 (FVD eFluor506) to exclude dead cells from analysis. CFSE geometric mean was further collected for cells in specified gates. Presented data was analyzed using FlowJo software.

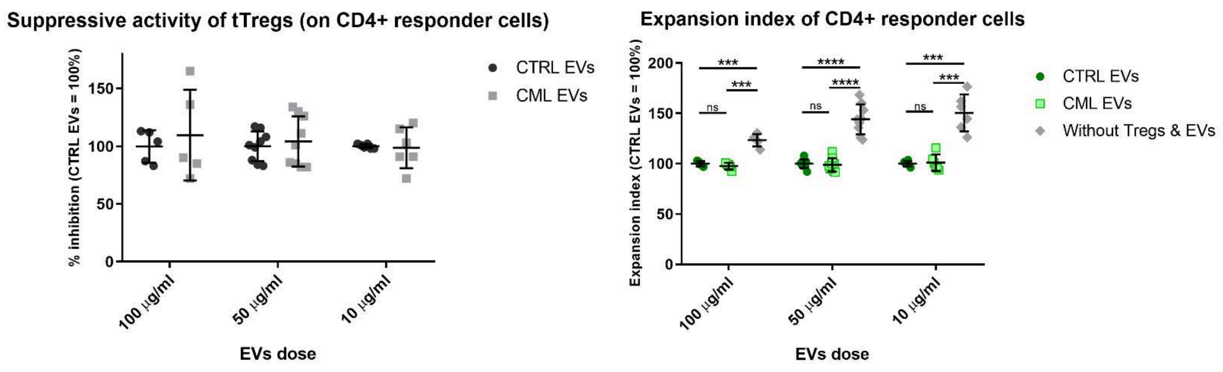

Supporting Information Figure 5. CML-derived EVs do not influence suppressive activity of tTregs towards CD4+ responder cells.

A) Suppressive activity (left) of regulatory T cells towards CD4+ responder cells - after treatment with different doses of CTRL and CML EVs. Expansion index (right) of CD4+ responder cells in culture with tTregs treated with different doses of CTRL and CML EVs. Data from 3 independent experiments (2-3 technical replicates each) were normalized to the average values in samples treated with CTRL EVs. Single data points with mean ± SD are presented. ***p<0.001, **** p<0.0001 (unpaired t test with Welch's correction).

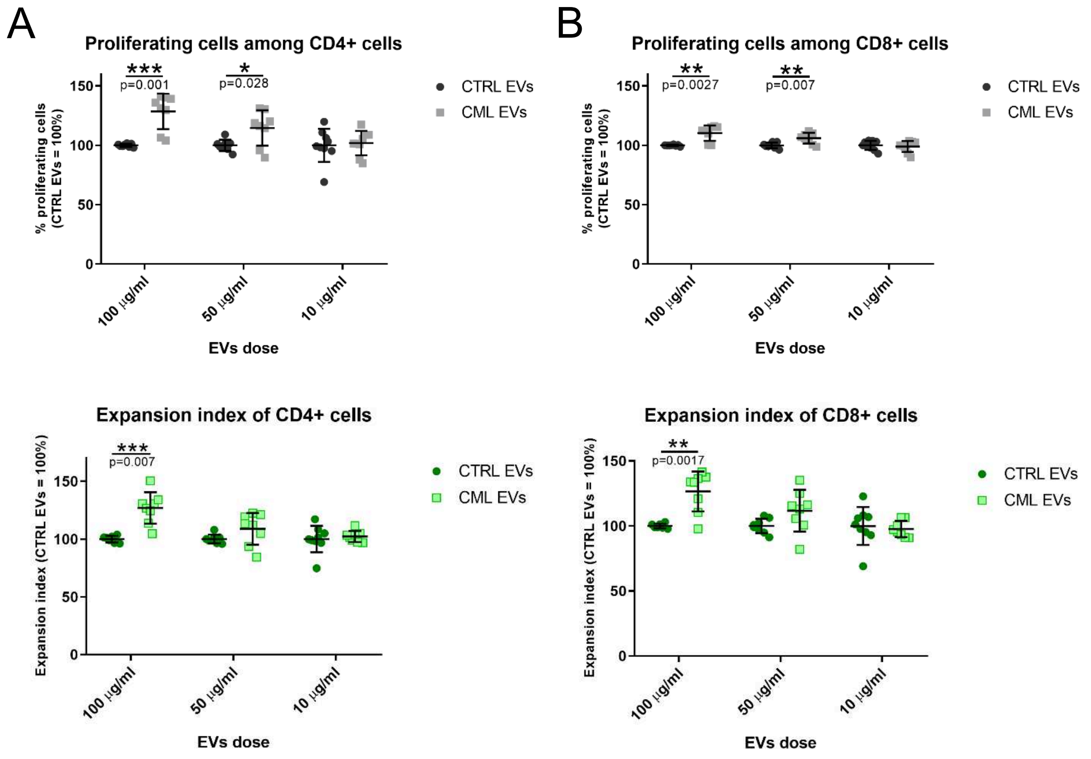

Supporting Information Figure 6. CML-derived EVs increase proliferation of CD4+ and CD8+ cells alone (in culture without tTregs).

A) Percentage of proliferating CD4+ cells (upper panel) and expansion index of CD4+ cells (lower panel) in culture of lymphocytes with CTRL and CML-derived EVs, but without tTregs. Data from 3 independent experiments (2-3 technical replicates each) were normalized to the average values in samples treated with CTRL EVs. B) Percentage of proliferating CD8+ cells (upper panel) and expansion index of CD8+ cells (lower panel) in culture of lymphocytes with CTRL and CML-derived EVs, but without tTregs. Data from 3 independent experiments (2-3 technical replicates each) were normalized to the average values in samples treated with CTRL EVs. Single data points with mean ± SD are presented. *$p<0.05$, ** $p<0.01$, *** $p<0.001$ (unpaired t test with Welch's correction).

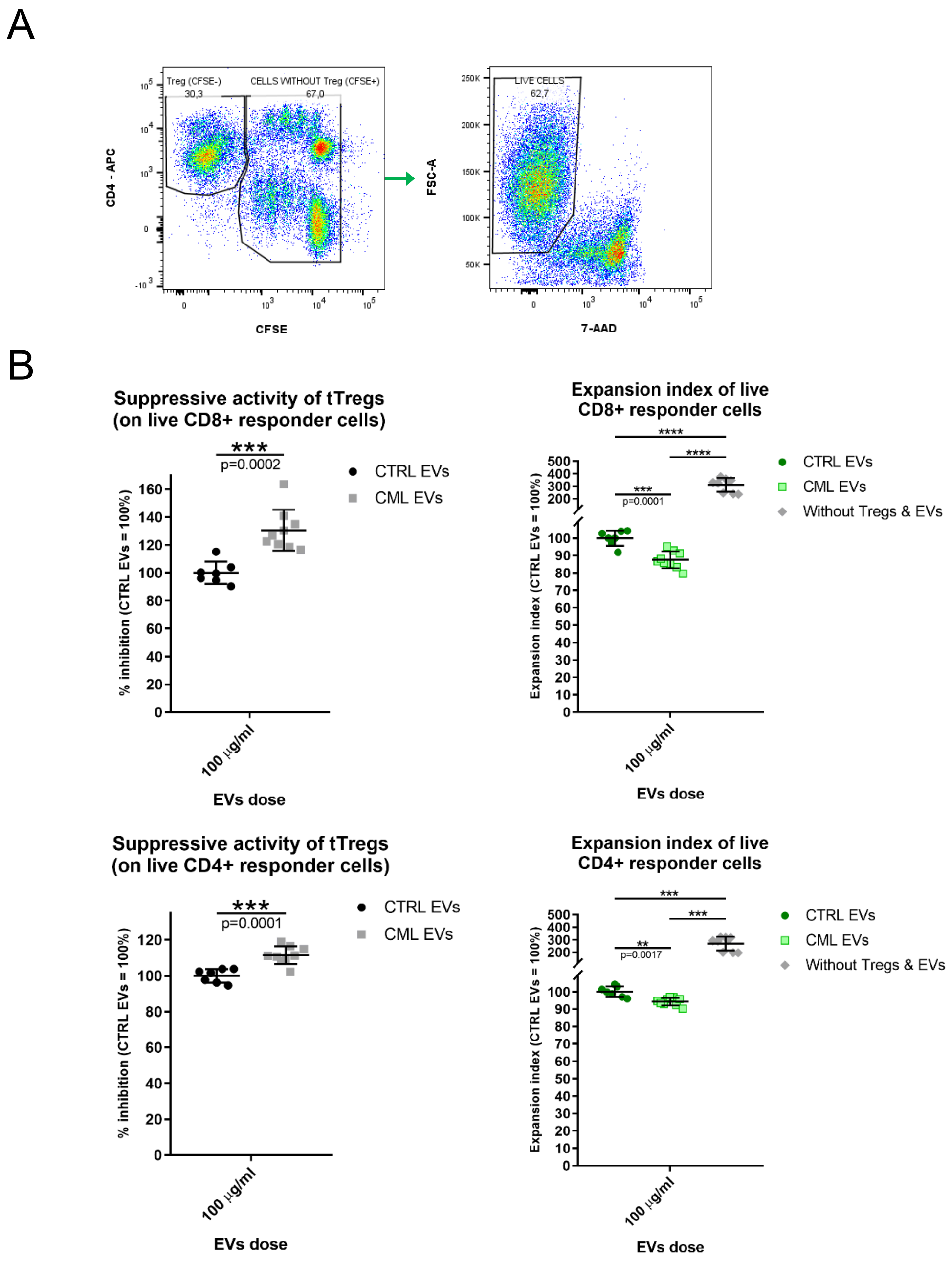

Supporting Information Figure 7. Analysis of suppression assay data (Fig. 1C) on live responder cells only.

A) Additional viability gate (based on 7-AAD dye) for analysis of suppression assay data (Fig. 1C). B) Suppressive activity (left) of tTregs and expansion index (right) of only live responder cells in a combined culture of tTregs, responder lymphocytes and CML/CTRL EVs. Data from 3 independent experiments (2-3 technical replicates each) were normalized to the average values in samples treated with CTRL EVs. Single data points with mean ± SD are presented. **p<0.01, ***p<0.001, ****p<0.0001 (unpaired t-test with Welch's correction).

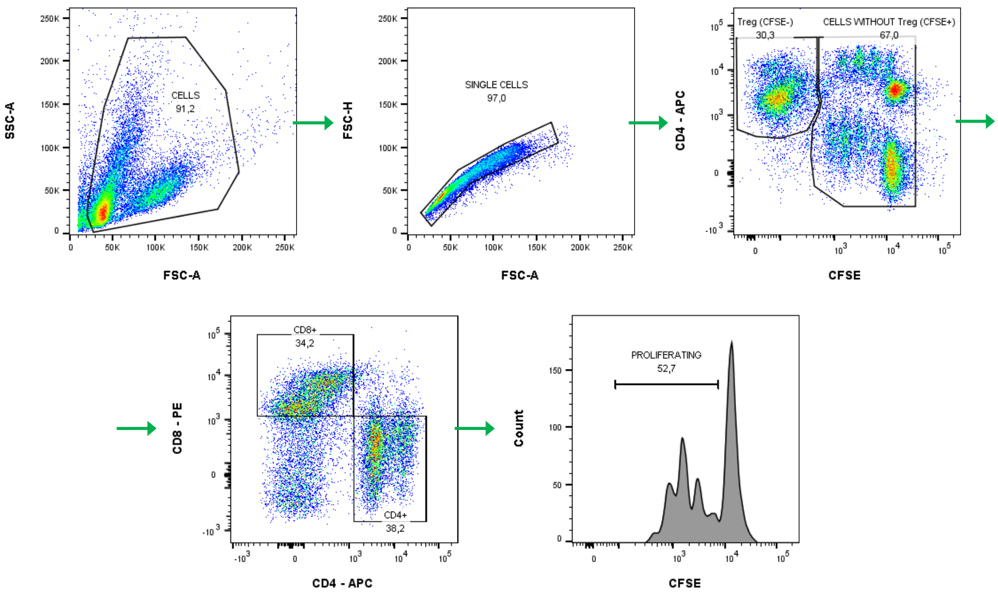

Supporting Information Figure 8. Gating strategy for analysis of proliferation of responder cells in a suppression assay.

Representative demonstration (of 9 independent experiments) of the gating strategy for flow cytometric analysis of responder cells proliferation from in vitro cell culture. Cells collected from *in vitro* cultures were stained with fluorochrome-conjugated antibodies against CD4 (APC) and CD8 (PE or PerCP). Responder cells were distinguished from tTregs based on positive CFSE fluorescence. CFSE fluorescence was used to detect cell divisions. In some experiments, Cell Proliferation Dye eFluor450 was used instead of CFSE. Presented data was analyzed using FlowJo software.

A

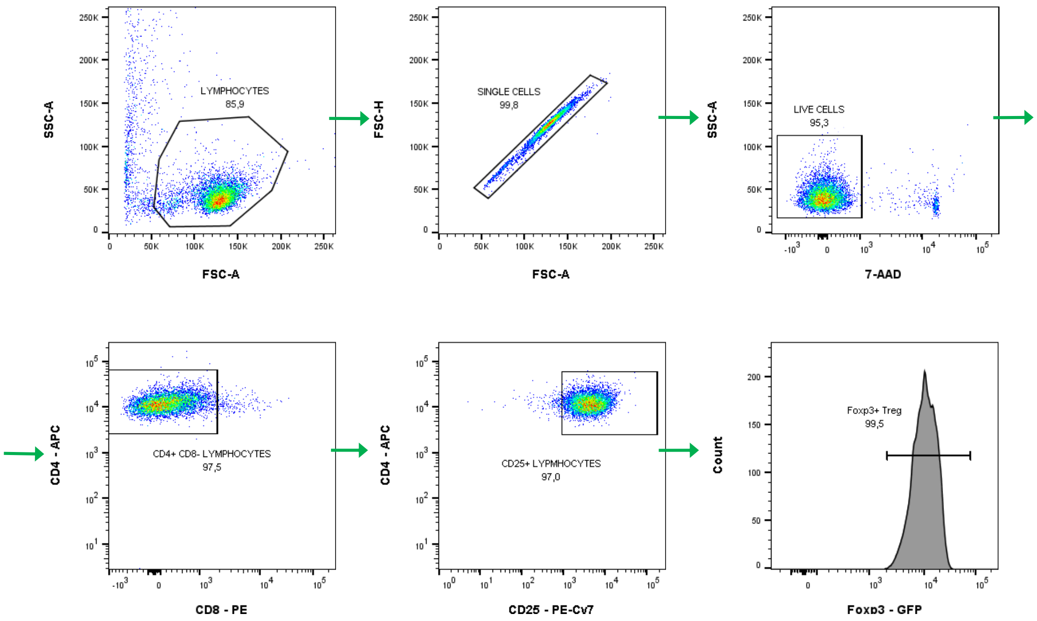

B

Negative control for Foxp3-GFP fluorescence in tTregs: tTreg from wild-type (C57BL/6) mice were sorted and cultured simultaneously with tTreg from B6.Cg-Foxp3^{tm2Tch} mice

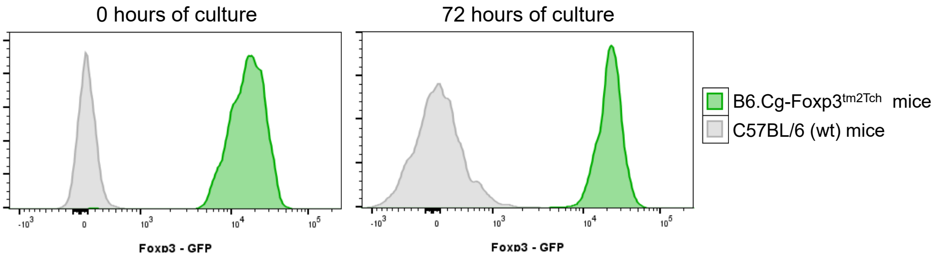

Supporting Information Figure 9. Gating strategy for analysis of Foxp3 level in sorted tTregs.

A) Representative demonstration (of 4 independent experiments) of the gating strategy for flow cytometric analysis of Foxp3 level in tTreg cultured *in vitro*. Cells collected from in vitro cultures were stained with fluorochrome-conjugated antibodies against CD4 (APC), CD8 (PE), CD25 (PE-Cy7) and with 7-AAD dye to exclude dead cells from analysis. Foxp3 level was determined based on GFP signal (as cells were sorted from B6.Cg-Foxp3^{tm2Tch} transgenic mice that co express Foxp3 and EGFP) among cells in the “Foxp3+ Treg” gate. Presented data was analyzed using FlowJo software. B) Negative control for Foxp3-GFP fluorescence in sorted tTregs – comparison of GFP fluorescence in tTregs from C57BL/6 (WT) and B6.Cg-Foxp3^{tm2Tch} (Foxp3-EGFP) mice, after sorting and after 72 hours of *in vitro* culture.
